# Invasive Infections Caused by Lancefield Groups C/G and A *Streptococcus*, Western Australia, Australia, 2000–2018

**DOI:** 10.3201/eid2811.220029

**Published:** 2022-11

**Authors:** Cameron M. Wright, Rachael Moorin, Glenn Pearson, John Dyer, Jonathan Carapetis, Laurens Manning

**Affiliations:** Curtin University, Perth, Western Australia, Australia (C.M. Wright, R. Moorin);; University of Tasmania, Hobart, Tasmania, Australia (C.M. Wright);; The University of Western Australia, Crawley, Western Australia, Australia (C.M. Wright, R. Moorin, J. Carapetis, L. Manning);; Fiona Stanley Fremantle Hospitals Group, Murdoch, Western Australia, Australia (C.M. Wright, J. Dyer, L. Manning);; Telethon Kids Institute, University of Western Australia, Perth (G. Pearson, J. Carapetis, L. Manning);; Perth Children’s Hospital, Perth (J. Carapetis)

**Keywords:** Group C *Streptococcus*, Group G *Streptococcus*, *Streptococcus*, infectious diseases, public health, Lancefield groups, Australia, bacteria

## Abstract

Epidemiologic data on invasive group C/G *Streptococcus* (iGCGS) infections are sparse internationally. Linked population-level hospital, pathology, and death data were used to describe the disease burden in Western Australia, Australia, during 2000–2018 compared with that of invasive group A *Streptococcus* (GAS, *Streptococcus pyogenes*) infections. Of 1,270 cases, 866 (68%) occurred in men. Patients with iGCGS infection were older (median age 62 years) than those with invasive GAS (median age 44 years; p<0.0001). The age and sex-adjusted incidence rate ratio by year was 1.08 (95% CI 1.07–1.09). The incidence rate ratio for Indigenous compared with non-Indigenous Australians was 3.6 (95% CI 3.0–4.3). The all-cause 90-day death rate was 9% for iGCGS infection compared with 7% for invasive GAS (p = 0.03). iGCGS infection was more common in men and older persons and had a higher death rate, perhaps reflecting the effect of age and comorbidities on incidence and death.

Invasive, β-hemolytic *Streptococcus* disease is associated with high rates of illness and death and substantial financial cost (*1*–*3*). Human pathogenic β-hemolytic *Streptococcus* include Lancefield groups A (GAS, *Streptococcus pyogenes*), B (GBS, *S. agalactiae*), and C and G (GCGS, multiple species) (*4*). An understanding of the population-level disease burden is essential for planning preventive and management strategies (*2*). For GCGS, the major subspecies causing human infection is *S. dysgalactiae* subspecies *equisimilis*, which shares virulence factors with GAS, including the M protein (*2*). Evidence from epidemiologic studies and animal models also link GCGS infection to postinfectious immunologic complications such as rheumatic heart disease (*5*,*6*). For these reasons, GCGS and GAS have overlapping clinical manifestations and, from a prevention perspective, development efforts toward a vaccine for GAS may have off-target effects in preventing GCGS infection (*7*).

Compared with knowledge about invasive GAS infections, much less is known about the epidemiology of invasive GCGS (iGCGS) disease or how clinical features and outcomes differ between them. Unlike GAS, which is notifiable in many jurisdictions, such as the United Kingdom (*8*) and Canada (*9*), iGCGS is not considered a notifiable disease in any jurisdiction. For the few settings where comparative epidemiologic data are available, the incidence of iGCGS infection is similar or higher than for invasive GAS (*10*,*11*). Also shared in common with invasive GAS infections, iGCGS has a death rate of 5%–10%, and its incidence appears to be increasing in recent years in some countries (*10*,*12*–*14*).

In a recent study, we demonstrated an increasing incidence of invasive GAS infection that disproportionately affects Indigenous Australians (*15*). By using this same methodology, we sought to describe the epidemiology of iGCGS infection in terms of incidence, median length of hospital stay, and all-cause deaths and to compare these metrics with those in patients with invasive GAS infections.

## Methods

Reporting was based on the RECORD (REporting of studies Conducted using Observational Routinely collected health Data) statement (*16*). Ethics approval was provided by the Western Australia (WA) Department of Health Human Research Ethics Committee (HREC) (#2019/03) and the WA Aboriginal Health Ethics Committee (#899). The University of WA HREC acknowledged external approval by the WA Department of Health HREC (RA/4/20/5695). A consent waiver was granted, meaning individual consent was not required. This study was designed as a population-based data linkage study.

### Setting

WA is the largest state in Australia, covering about one third of the continent (2.6 million km^2^). In 2018, the population was 2.6 million persons (10% of the population of Australia), including ≈2 million persons living in the capital city, Perth (*17*); 4% of the population were Indigenous persons (*18*). The climate ranges from tropical in the north to desert in the central regions and temperate in the south. The climate of the Kimberley and Pilbara regions is tropical, and the proportion of Indigenous persons is higher there than in other regions of WA (in 2016, accounting for 42% of the population in Kimberley and 14% in Pilbara) (*19*). Regional, climate-based, and demographic variation in health-related variables can accordingly be explored in WA, as we did in our previous investigation of lower leg cellulitis, by using linked hospital and emergency department data (*20*,*21*).

### Data Sources and Measurement

The WA Data Linkage System used best-practice methods (*22*) to link the Hospital Morbidity Data Collection (HMDC), consisting of all WA public and private hospital records; PathWest, the government-owned pathology services provider for metropolitan and regional public hospitals; and death registrations, which are used in state-level and national-level death statistics. Linked data had a scrambled unique identifier for each person.

### Case Definition

We analyzed data for cases of iGCGS and invasive GAS infection occurring during January 1, 2000–December 31, 2018, among WA residents. Methods relating to invasive GAS infections are described elsewhere (*15*). In brief, microbiologically confirmed cases were defined as GCGS isolated from a normally sterile site (blood, cerebrospinal fluid, or other normally sterile fluid or sterile tissue) identified in PathWest laboratory data. Group C and G *Streptococcus* were grouped together as GCGS as characterized in the PathWest data. Diagnoses of iGCGS infection were identified in the HMDC by principal diagnostic codes from the International Classification of Diseases, 10th Revision, Australian Modification (ICD-10-a.m.) for GCGS-specific invasive disease (Appendix Table 1), referred to hereafter as the HMDC cohort definition. To be included, a case had to be accompanied by the ICD-10-a.m. code for GCGS infection (B95.41, Streptococcus, group C, as the cause of diseases classified to other chapters; or B95.42, Streptococcus, group G, as the cause of diseases classified to other chapters) as the first additional diagnosis, without any ICD-10-a.m. codes for other bacterial infection diagnoses. We included all cases of iGCGS infection according to PathWest or HMDC cohort definitions (or both) in our analysis.

Pathology data were grouped within a hospitalization (regardless of whether the hospitalization fulfilled the HMDC cohort definition) if collection was within 2 days of an admission date, thus enabling previous outpatient clinic or emergency department specimen collection. The record date for a case was the first collection date for each episode or the admission date if there was no confirmatory isolate. Because iGCGS infection is an acute condition, persons could have incident disease more than once, but all records for a person occurring within 30 days were considered a single event (*23*). We also conducted a sensitivity analysis restricting to 1 case per person only.

### Case Characteristics

Age, sex, hospital admission and separation (discharge) dates, diagnostic codes, region of residence, census-specific remoteness of residence area (major cities, inner regional, outer regional, remote, or very remote) (*24*), census-specific postcode-based values according to socioeconomic index for relative social disadvantage (separated into quintiles) (*25*), and admission to an intensive care unit were extracted from the HMDC. Indigenous status was provided as part of the linkage process. Date of death was extracted from death registrations. Region of residence was divided into tropical (Kimberley and Pilbara regions) and nontropical and into metropolitan and regional. PathWest data included the unique patient identifier code, sample collection date, and isolation site. For cases identified through PathWest only, we sourced relevant demographic data (e.g., age, sex) from the HMDC, because all hospital records for the cohort were available. Case-patients were designated as a WA resident, Indigenous, or living in a tropical area (not mutually exclusive) if assigned one of these designations for any part of a case.

### Statistical Analyses

#### Descriptive Statistics

We included cases with missing demographic data but did not include them in analyses stratified by the missing variable. For statistical comparisons of categorical variables, we used a χ^2^ test and a nonparametric equality of medians test for continuous variables. We explored seasonality as wet (November–April) and dry (May–October) seasons for tropical and 4 seasons for nontropical regions.

#### Crude and Age-Standardized Incidence

We included only WA residents in incidence calculations and sourced midyear population denominators from the Australian Bureau of Statistics census estimates and projections (*17*). We calculated crude incidence stratified by sex, age group, Indigenous status (from 2001, owing to readily publicly available denominators) and region (from 2001, for the same reason). We calculated direct age-standardized annual incidence standardizing to the age-based population structure of cases in 2000. We also conducted subanalyses for cases in which GCGS was isolated only from blood or tissue culture. We performed negative binomial regression, selected because of overdispersed data, for incidence adjusting for year, age group, and sex. We also used negative binomial regression to model incidence adjusting for Indigenous status and year and separately by Indigenous status adjusting for year.

#### Median Length of Stay and All-Cause Deaths

For median length of stay, we treated interhospital transfers as a single admission. We calculated all-cause death at 30 days and 90 days after record date. We assessed differences in 30-day deaths by Indigenous status by using age group–adjusted and sex-adjusted logistic regression. We performed all analyses by using Stata SE version 14.0 (StataCorp LLC, https://www.stata.com).

## Results

### Demographic and Clinical Characteristics of Patients with iGCGS Infection

A total of 1,270 cases occurred during the study period. Of those, 1,195 (94%) were confirmed with PathWest microbiological data. Only 112 (9%) had an iGCGS-specific HMDC discharge diagnosis; of those, 37 (33%) had both HMDC-confirmed cases and microbiologic confirmation. This percentage was higher for public hospital cases (41%, 33/80) than for private hospital cases (13%, 4/32; p = 0.003). Of microbiologically confirmed cases, GCGS was isolated from blood in 713/1,195 (60%) cases, from tissue in 388 (32%) cases, and from other sterile fluids in 208 (17%) cases, although these types were not mutually exclusive. The most frequent principal diagnoses for hospitalizations with a corresponding GCGS isolate (i.e., samples within 2 days of admission) were other streptococcal sepsis (151 [12%]), cellulitis of other parts of limb (118 [9%]), and type 2 diabetes with foot ulcer with multiple causes (71 [6%]).

More than two thirds of cases were in men (866/1,270 [68%]; p<0.001) (Table), and the median age was 62 years (interquartile range [IQR] 47–75 years). Only 13 (1%) of 1,270 cases occurred in persons who were <14 years of age (Table). A total of 148 cases (12%) occurred in Indigenous Australians and 91 case-patients (7%) were from a tropical region of the state. Just over two thirds of cases were among persons from a major city (855/1,270 [67%]), and nearly one third of cases (384/1,270 [30%]) were in the most disadvantaged quintile in the index for relative social disadvantage. Seasonality was not evident among nontropical cases: 306/1,175 cases occurred during summer (26%), 298 occurred in autumn (25%), 268 occurred in winter (23%), and 303 occurred in spring (26%). In the tropics, more cases occurred in the wet season (59%) than in the dry season (41%; p = 0.07).

**Table:** Descriptive statistics of invasive group C/G *Streptococcus* disease, Western Australia, Australia, 2000–2018*

Characteristic	No. (%)	
Total	1,270	
Sex		
F	400 (31)	
M	866 (68)	
Missing/unknown	<5	
Age group, y		
<1	5 (0)	
1–4	<5	
5–14	6 (0)	
15–24	52 (4)	
25–34	80 (6)	
35–44	133 (10)	
45–54	181 (14)	
55–64	222 (17)	
65–74	244 (19)	
75–84	215 (17)	
>85	126 (10)	
Missing/unknown	<5	
Indigenous status		
Non-Indigenous	1,067 (84)	
Indigenous	148 (12)	
Missing/unknown	55 (4)	
Region of occurrence		
Nontropical	1,175 (93)	
Tropical	91 (7)	
Missing/unknown	<5	
Remoteness		
Major cities	855 (67)	
Inner regional	92 (7)	
Outer regional	128 (10)	
Remote	81 (6)	
Very remote	59 (5)	
Missing/unknown	55 (4)	
Socioeconomic status		
Most disadvantaged	384 (30)	
More disadvantaged	280 (22)	
Moderately disadvantaged	220 (17)	
Less disadvantaged	184 (14)	
Least disadvantaged	198 (16)	
Missing/unknown	<5	
30-d all-cause deaths	85 (7)	
90-d all-cause deaths	114 (9)	

*Categories with <5 patients are shown without accompanying percentage values to comply with confidentiality data requirements. Some rows do not sum to 100 because of this or because of rounding. Analogous data for invasive group A *Streptococcus* disease has been published separately (*15*).

### Crude and Age-Standardized Incidence

**Figure 1:**
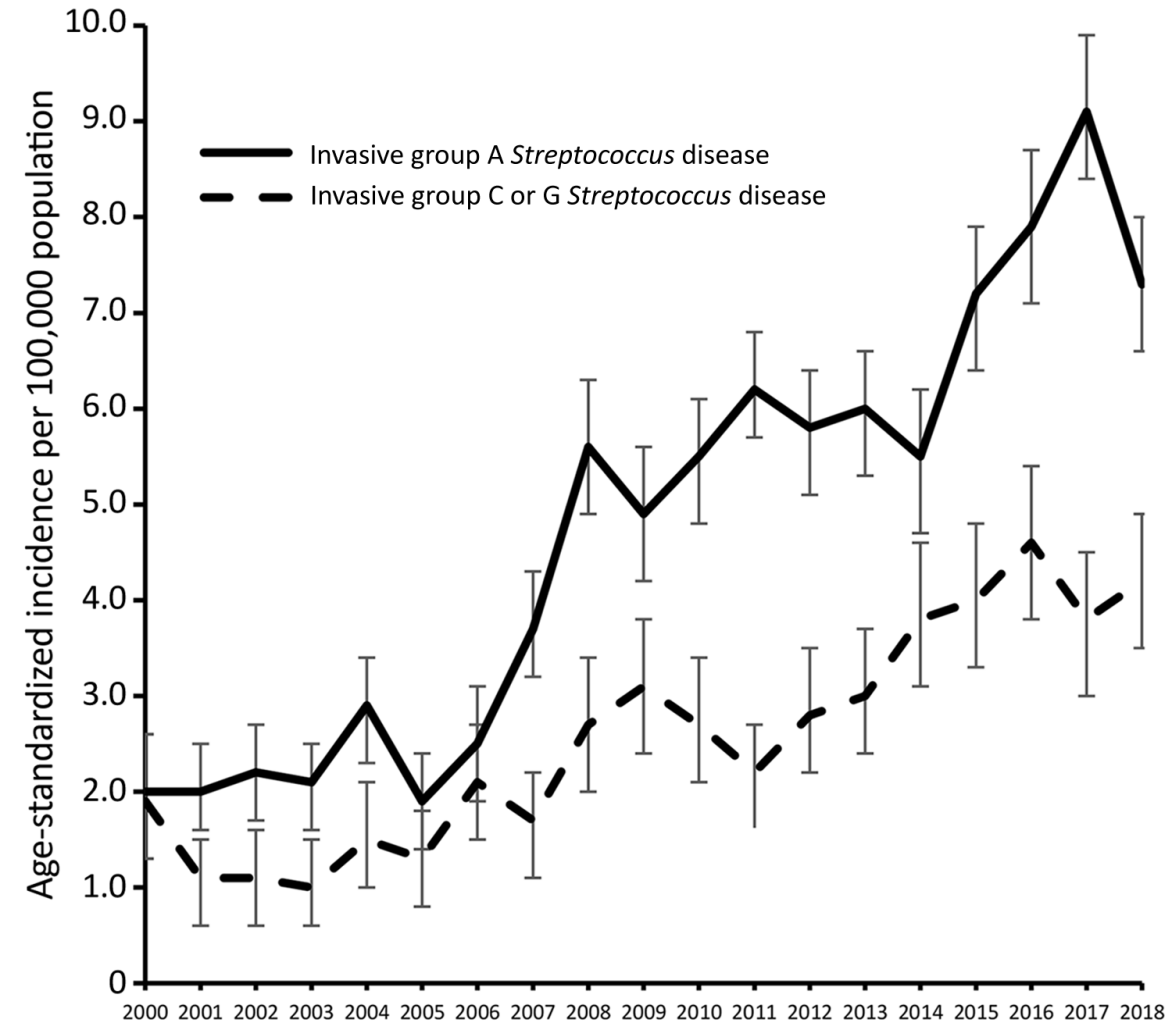
Age-standardized incidence of invasive group A and C/G *Streptococcus* disease, Western Australia, Australia, 2000–2018. The baseline age distribution is the year 2000. Error bars indicate 95% CI.

**Figure 2:**
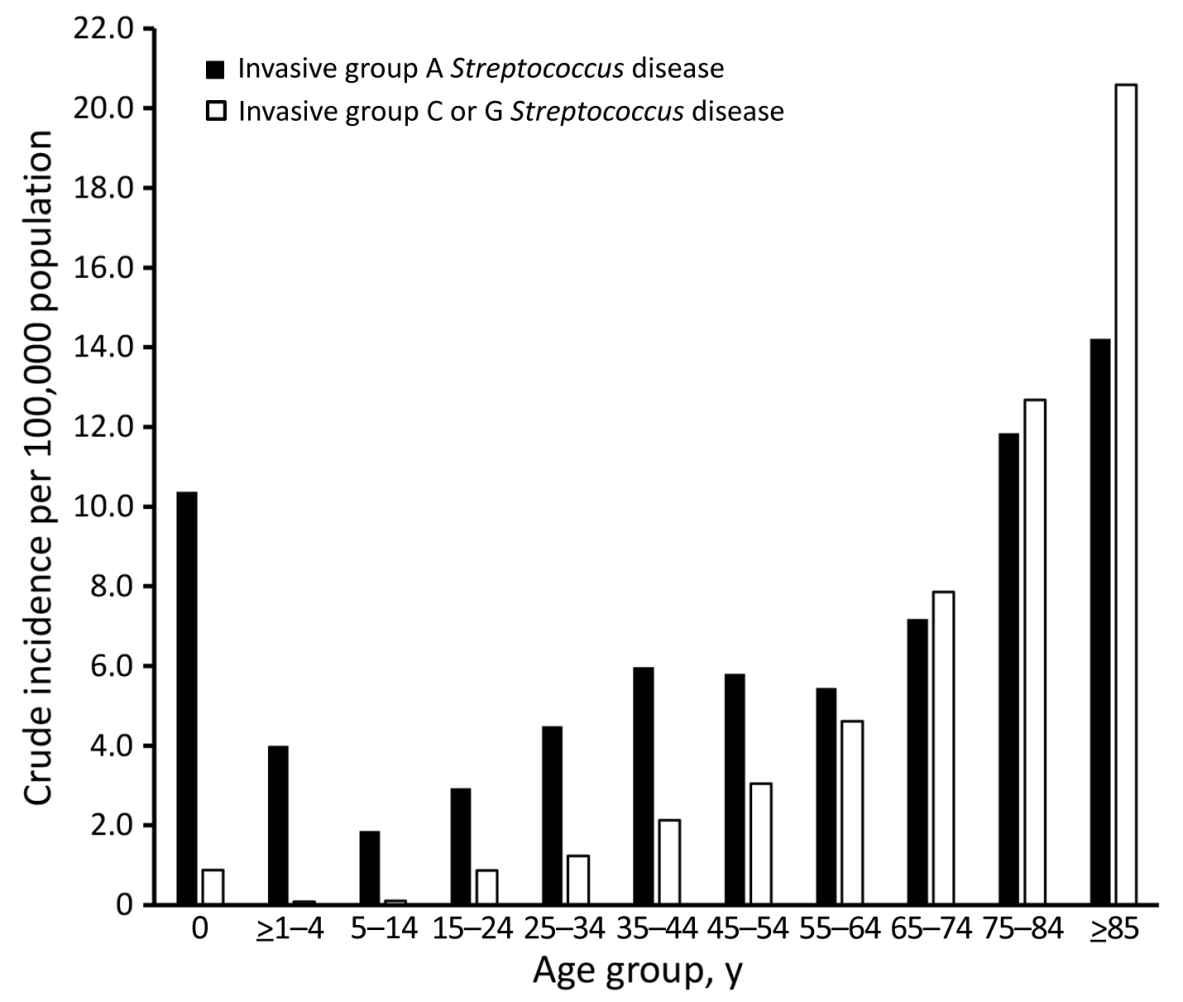
Age group distribution of invasive group A and C/G *Streptococcus* disease, Western Australia, Australia, 2000–2018.

The age-standardized incidence of iGCGS disease increased from a low of 1.0 (95% CI 0.5–1.4) cases/100,000 population in 2003 to a peak of 4.6 (95% CI 3.8–5.4 cases) cases/100,000 population in 2017 (Figure 1). The adjusted incidence rate ratio (IRR) for year of diagnosis since 2000 was 1.08 (95% CI 1.07–1.09). A sensitivity analysis restricted to 1 case per person (76 [6%] had >1 iGCGS infection) did not show any difference in incidence, compared with analysis allowing repeat infection separated by >30 days (Appendix Figure 1). The numbers of incident cases with blood or tissue isolates each increased over time (Appendix Figure 2). The age-group based crude incidence of iGCGS disease increased with age (Figure 2).

**Figure 3:**
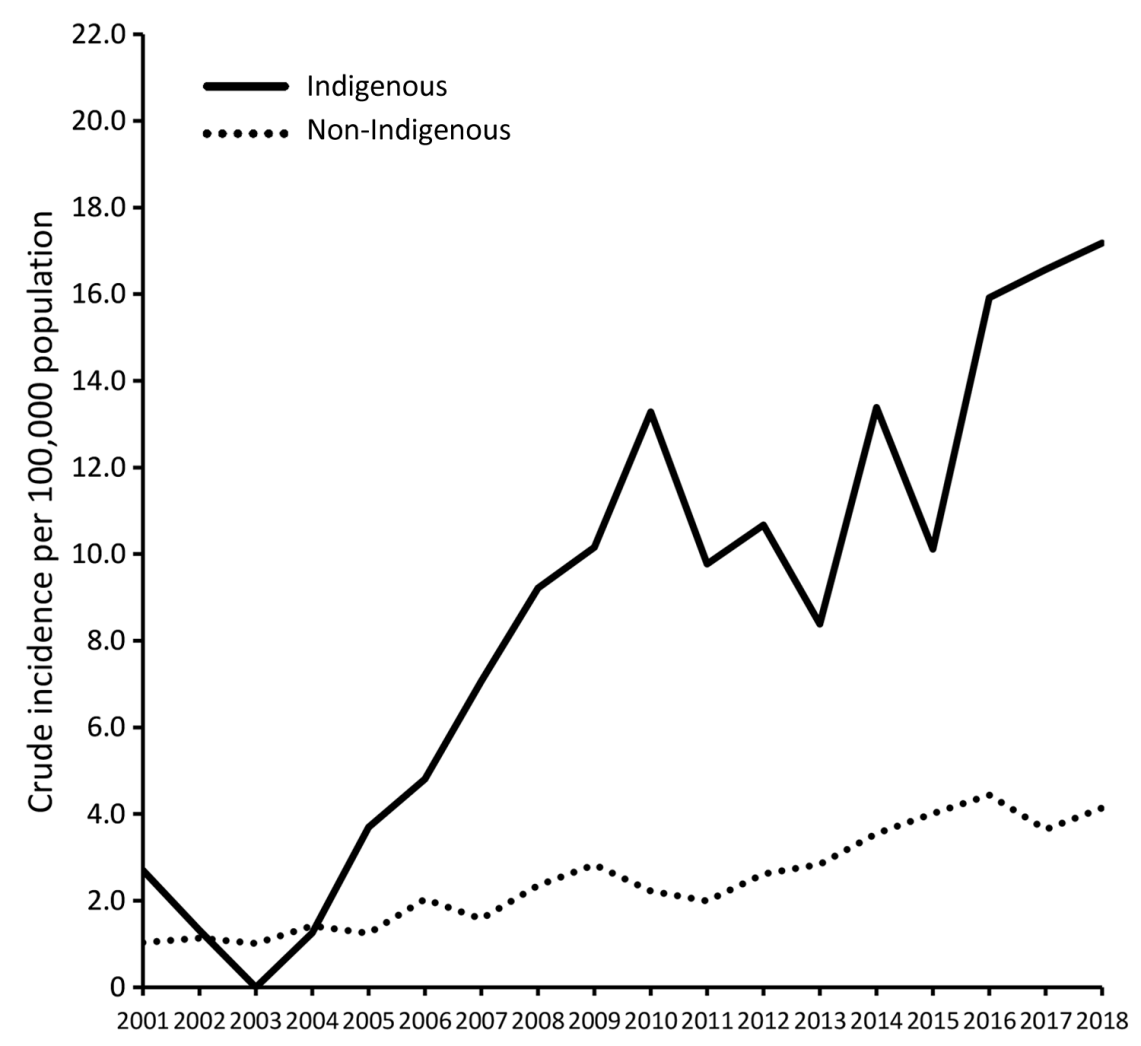
Indigenous versus non-Indigenous distribution of invasive group C/G *Streptococcus* disease, Western Australia, Australia, 2000–2018.

Crude incidence increased over time for Indigenous persons (per year: IRR 1.11, 95% CI 1.07–1.15) and non-Indigenous persons (IRR 1.09, 95% CI 1.07–1.10). Crude incidence was higher for Indigenous persons than for non-Indigenous persons from 2004 on, peaking in 2018 at 17.2 (95% CI 9.2 –25.1) cases/100,000 population in Indigenous persons and 4.1 (95% CI 3.3–4.9) cases/100,000 population in non-Indigenous persons (Figure 3). The year-adjusted IRR comparing Indigenous and non-Indigenous Australians was 3.6 (95% CI 3.0–4.3). Crude incidence was higher for Indigenous persons in both metropolitan and regional areas (data not shown). Incidence was consistently higher among men than women (adjusted IRR 2.3, 95% CI 2.1–2.6) (Appendix Figure 3).

### Median Length of Stay and All-Cause Death

Median length of stay was 10 days (IQR 2–24 days). In 85 (7%) cases, the patient had died of any cause by 30 days, and 114 (9%) patients had died by 90 days (Table). All-cause death was lower at 30 days for Indigenous patients (4/148 cases [3%]) than non-Indigenous patients (81/1,067 [8%]), but the difference was not statistically significant after adjustment for age group and sex (adjusted odds ratio 0.7, 95% CI 0.3–1.4). At 30 days, the all-cause death rate was higher in cases in which GCGS was isolated from blood (92/713 [13%]) than in cases with no blood isolate (22/557 [4%]; p<0.001) and higher if the patient was admitted to intensive care (9/55 [16%]) than if not (76/1,215 [6%]; p<0.001). In cases involving patients >85 years of age, the 90-day all-cause death rate was 32%.

### Comparison with Invasive GAS Infection

The incidence of invasive GAS infection was higher than for iGCGS disease over the study period but increased at a similar annual rate; the yearly IRR was 1.09 (Figure 1). Visual assessment of the age distribution (Figure 2) indicates a higher concentration of iGCGS disease in older age groups compared with invasive GAS disease. Compared with patients with invasive GAS infections, patients with iGCGS were older (median 62 [IQR 47–75] vs. 44 [IQR 29 – 62] years; p<0.0001). The percentage of men with invasive GAS disease was lower than the percentage of men with iGCGS disease (57% vs. 68%; p<0.001). Conversely, the proportion of cases among Indigenous persons was higher for invasive GAS than for iGCGS (34% vs. 12%; p<0.001).

The median length of stay was also higher for iGCGS patients than for invasive GAS patients (10 [IQR 2–24] vs. 7 [IQR 3–16] days; p<0.0001). The 30-day all-cause death rate was higher for patients with invasive GAS disease than for those with iGCGS disease (Appendix Table 2), but this difference was not significant (p = 0.06). The 90-day death rate for iGCGS was higher than that for invasive GAS disease (9% [114/1,270] vs. 7% [156/2,237]; p = 0.03). However, the age-adjusted odds of 90-day death were higher for invasive GAS disease than for iGCGS disease (adjusted odds ratio 1.33, 95% CI 1.02–1.73).

## Discussion

These data show an increasing incidence of iGCGS infections over time in WA. Cases occurred predominately among older persons and men, and the all-cause 90-day death rate among infected persons was high. As with invasive GAS, the incidence of iGCGS among Indigenous Australians was higher than among non-Indigenous Australians, although the respective IRRs over the study period were similar (1.11 and 1.09). The increase in iGCGS disease in WA is a critical finding, because development of a GAS vaccine could benefit the older population affected more commonly by iGCGS infection, if there are off-target protective effects across Lancefield groups (*7*).

In a 2017 study using similar methodology and dataset, we demonstrated that the combined incidence of iGCGS was approximately half that of invasive GAS disease (*15*). Similarly, although both diseases were more common among Indigenous than among non-Indigenous Australians, the relative risk was higher for invasive GAS (IRR 13.1) (*15*) than for iGCGS (IRR 3.6). Compared with findings for other settings, the reported incidence in this study (4.6 cases/100,000 population) was lower than that in a study from southern Hungary (11 cases/100,000 population) (*12*) but higher than that reported in the United States (2.8 cases/100,000 population) (*26*).

In the context of Australia, these data extend previous work from North Queensland (*27*). Harris et al. (*27*) reported on GCCS bacteremia in North Queensland during 1996–2009, finding two thirds of group G cases occurred in men. Our data are in accordance with this finding; 68% of cases occurred in men. Harris et al. (*27*) found a 28-day all-cause death rate of 5.5% (5/91) for GCCS bacteremia, lower than the 9% rate in our study, although with a much smaller denominator. The mean age of patients with GCCS bacteremia reported by Harris et al. (*27*) was 43 years, younger than those in this study.

The age distribution of iGCGS infection differs from invasive GAS disease (*15*). Unlike GAS, which occurs most frequently in older and younger age groups but also occurs in the middle age groups, iGCGS is characteristically an infection of middle-aged and older persons. Age is likely a surrogate marker for comorbidities such as diabetes (*28*), alcohol use, and liver diseases, which have been reported elsewhere as risk factors for iGCGS infection (*10*).

Factors predisposing persons to iGCGS disease might also contribute to the high observed all-cause death rate and prolonged length of hospital stay reported in this study. To place all-cause deaths in context, the 30-day death rate among those >85 years of age at diagnosis was much higher (25%) than the rate of ≈10% for this age group after neck of femur fracture (*29*). When compared with invasive GAS disease, the 30-day death rate in older age groups (>65 years of age) was similar to or marginally lower than that for patients with iGCGS disease (Appendix Table 2), indicating that the differences in overall death proportions for each is driven primarily by age.

Although it serves most public hospitals, PathWest is not the sole pathology provider in WA. For this reason, we augmented laboratory data with hospital records, including 75 cases (6%) without laboratory confirmation. The percentage of microbiologically confirmed HMDC cases was larger for public hospitals than private hospitals, which suggests that HMDC cases without PathWest confirmations were probably confirmed by private microbiology providers. The conservative diagnostic definition for HMDC cases minimized the effect of any HMDC coding errors. However, because of the requirement for a GCGS-specific descriptor as an additional diagnostic code (B95.41 or B95.42), some underreporting of cases confirmed by an alternative microbiology service provider could have occurred. Multiple potential drivers for the increase in iGCGS disease exist, such as changing risk factor prevalence (e.g., immunocompromise or immune senescence) (*30*,*31*) and changing demographics (e.g., an aging population). However, the similar increases in iGCGS infection and invasive GAS disease make system-level factors a probable contributor. These factors could include increased blood culture and other usually sterile specimen collection, enabling detection of previously undetected infections; increased referral of specimens from private laboratories to PathWest; or improved capture in routinely collected data. Repeat surveillance will be useful for monitoring contemporary trends in disease burden. We did not perform adjustment of all-cause deaths for chronic medical conditions because the comorbidity information was limited to that coded as relevant for individual hospital stays. The administrative data we analyzed were not collected for research purposes, but the use of these data in clinical practice (PathWest), clinical activity reporting (HMDC), and for informing national mortality data (death registrations) meant that the linked data analysis was appropriate for our study.

The incidence of iGCGS disease in WA increased during 2000–2018; cases occurred predominately among older persons and men, and Indigenous persons were at increased risk. This infection was marked by high all-cause death within 30 and 90 days, especially among the elderly, and a prolonged length of hospital stay. Although further research should assess the contribution of such comorbidities as diabetes to inform preventive efforts, these data highlight that iGCGS infection has been a neglected pathogen of older persons and that Indigenous persons face a higher risk for infection.

Appendix Additional information about invasive infections caused by Lancefield groups C/G and A *Streptococcus*, Western Australia, Australia, 2000–2018

## References

[1] Hughes GJ, VAN Hoek AJ, Sriskandan S, Lamagni TL. The cost of hospital care for management of invasive group A streptococcal infections in England. Epidemiol Infect. 2015;143:1719–30. 10.1017/S0950268814002489 25262779 PMC9507226

[2] Parks T, Barrett L, Jones N. Invasive streptococcal disease: a review for clinicians. Br Med Bull. 2015;115:77–89. 10.1093/bmb/ldv027 26209784

[3] Centers for Disease Control and Prevention. Case definitions for infectious conditions under public health surveillance. MMWR Recomm Rep. 1997;46(RR-10):1–55. 9148133

[4] Facklam R. What happened to the streptococci: overview of taxonomic and nomenclature changes. Clin Microbiol Rev. 2002;15:613–30. 10.1128/CMR.15.4.613-630.2002 12364372 PMC126867

[5] Sikder S, Williams NL, Sorenson AE, Alim MA, Vidgen ME, Moreland NJ, et al. Group G Streptococcus induces an autoimmune carditis mediated by interleukin 17A and interferon γ in the Lewis rat model of rheumatic heart disease. J Infect Dis. 2018;218:324–35. 10.1093/infdis/jix637 29236994

[6] Haidan A, Talay SR, Rohde M, Sriprakash KS, Currie BJ, Chhatwal GS. Pharyngeal carriage of group C and group G streptococci and acute rheumatic fever in an Aboriginal population. Lancet. 2000;356:1167–9. 10.1016/S0140-6736(00)02765-3 11030302

[7] Brandt ER, Sriprakash KS, Hobb RI, Hayman WA, Zeng W, Batzloff MR, et al. New multi-determinant strategy for a group A streptococcal vaccine designed for the Australian Aboriginal population. Nat Med. 2000;6:455–9. 10.1038/74719 10742155

[8] Public Health England. Notifiable diseases: annual report. Manchester: Public Health England; 2020 [cited 2020 Oct 6]. https://www.gov.uk/government/publications/notifiable-diseases-annual-report

[9] Government of Canada. National case definition: Invasive group A streptococcal disease. Ottawa: Government of Canada; 2019 [cited 2019 Mar 30]. https://www.canada.ca/en/public-health/services/diseases/group-a-streptococcal-diseases/health-professionals/national-case-definition.html

[10] Fujiya Y, Hayakawa K, Gu Y, Yamamoto K, Mawatari M, Kutsuna S, et al. Age-related differences in clinical characteristics of invasive group G streptococcal infection: Comparison with group A and group B streptococcal infections. PLoS One. 2019;14:e0211786. 10.1371/journal.pone.0211786 30845149 PMC6405256

[11] Babiker A, Li X, Lai YL, Strich JR, Warner S, Sarzynski S, et al. Effectiveness of adjunctive clindamycin in β-lactam antibiotic-treated patients with invasive β-haemolytic streptococcal infections in US hospitals: a retrospective multicentre cohort study. Lancet Infect Dis. 2021;21:697–710. 10.1016/S1473-3099(20)30523-5 33333013 PMC8084921

[12] Gajdács M, Ábrók M, Lázár A, Burián K. Beta-haemolytic group A, C and G streptococcal infections in southern Hungary: a 10-year population-based retrospective survey (2008–2017) and a review of the literature. Infect Drug Resist. 2020;13:4739–49. 10.2147/IDR.S279157 33408489 PMC7781025

[13] Kittang BR, Bruun T, Langeland N, Mylvaganam H, Glambek M, Skrede S. Invasive group A, C and G streptococcal disease in western Norway: virulence gene profiles, clinical features and outcomes. Clin Microbiol Infect. 2011;17:358–64. 10.1111/j.1469-0691.2010.03253.x 20456456

[14] Oppegaard O, Mylvaganam H, Kittang BR. Beta-haemolytic group A, C and G streptococcal infections in Western Norway: a 15-year retrospective survey. Clin Microbiol Infect. 2015;21:171–8. 10.1016/j.cmi.2014.08.019 25658557

[15] Wright CM, Moorin R, Pearson G, Dyer JR, Carapetis JR, Manning L. Increasing incidence of invasive group A streptococcal disease in Western Australia, particularly among Indigenous people. Med J Aust. 2021;215:36–41. 10.5694/mja2.51117 34091892

[16] Benchimol EI, Smeeth L, Guttmann A, Harron K, Moher D, Petersen I, et al.; RECORD Working Committee. The REporting of studies Conducted using Observational Routinely-collected health Data (RECORD) statement. PLoS Med. 2015;12:e1001885. 10.1371/journal.pmed.1001885 26440803 PMC4595218

[17] Australian Bureau of Statistics. Table 4. Estimated resident population, states and territories (Number). 2020 [cited 2020 Jan 8]. http://www.abs.gov.au/AUSSTATS/abs@.nsf/DetailsPage/3101.0Mar%202018?OpenDocument

[18] Australian Bureau of Statistics. Estimates and Projections, Aboriginal and Torres Strait Islander Australians [cited 2020 Jun 2]. https://www.abs.gov.au/AUSSTATS/abs@.nsf/mf/3238.0

[19] Government of Western Australia. Regional Population Data [cited 2020 Sep 17]. https://catalogue.data.wa.gov.au/dataset/western-australia-regional-population/resource/eeecaf08-55e3-4026-99fc-f98a383e2812

[20] Cannon J, Dyer J, Carapetis J, Manning L. Epidemiology and risk factors for recurrent severe lower limb cellulitis: a longitudinal cohort study. Clin Microbiol Infect. 2018;24:1084–8. 10.1016/j.cmi.2018.01.023 29427799

[21] Cannon J, Rajakaruna G, Dyer J, Carapetis J, Manning L. Severe lower limb cellulitis: defining the epidemiology and risk factors for primary episodes in a population-based case-control study. Clin Microbiol Infect. 2018;24:1089–94. 10.1016/j.cmi.2018.01.024 29427797

[22] Kelman CW, Bass AJ, Holman CD. Research use of linked health data—a best practice protocol. Aust N Z J Public Health. 2002;26:251–5. 10.1111/j.1467-842X.2002.tb00682.x 12141621

[23] Boyd R, Patel M, Currie BJ, Holt DC, Harris T, Krause V. High burden of invasive group A streptococcal disease in the Northern Territory of Australia. Epidemiol Infect. 2016;144:1018–27. 10.1017/S0950268815002010 26364646

[24] Australian Bureau of Statistics. The Australian Statistical Geography Standard (ASGS) remoteness structure [cited 2018 Oct 5]. https://www.abs.gov.au/websitedbs/d3310114.nsf/home/remoteness+structure

[25] Australian Bureau of Statistics. Socioeconomic indices for areas [cited 2018 Oct 15]. https://www.abs.gov.au/websitedbs/censushome.nsf/home/seifa

[26] Broyles LN, Van Beneden C, Beall B, Facklam R, Shewmaker PL, Malpiedi P, et al. Population-based study of invasive disease due to beta-hemolytic streptococci of groups other than A and B. Clin Infect Dis. 2009;48:706–12. 10.1086/597035 19187026

[27] Harris P, Siew DA, Proud M, Buettner P, Norton R. Bacteraemia caused by beta-haemolytic streptococci in North Queensland: changing trends over a 14-year period. Clin Microbiol Infect. 2011;17:1216–22. 10.1111/j.1469-0691.2010.03427.x 21073630

[28] Thomsen RW, Riis AH, Kjeldsen S, Schønheyder HC. Impact of diabetes and poor glycaemic control on risk of bacteraemia with haemolytic streptococci groups A, B, and G. J Infect. 2011;63:8–16. 10.1016/j.jinf.2011.05.013 21663970

[29] Moon A, Gray A, Deehan D. Neck of femur fractures in patient’s aged more than 85 years—are they a unique subset? Geriatr Orthop Surg Rehabil. 2011;2:123–7. 10.1177/2151458511414562 23569681 PMC3597318

[30] Shahin A, Saba M, Greene J. A retrospective chart review on the clinical characteristics and outcomes of cancer patients with group C, F, or G β-hemolytic streptococcal infections. Infect Dis Clin Pract. 2019;27:205–10. 10.1097/IPC.0000000000000723

[31] Lewthwaite P, Parsons HK, Bates CJ, McKendrick MW, Dockrell DH. Group G streptococcal bacteraemia: an opportunistic infection associated with immune senescence. Scand J Infect Dis. 2002;34:83–7. 10.1080/00365540110077209 11928858

