# Supplementary material for: Invasive Infections Caused by Lancefield Groups C/G and A Streptococcus, Western Australia, Australia, 2000–2018
Source: Emerg Infect Dis. 2022 Nov;28(11):2190–7. doi: 10.3201/eid2811.220029 (PMC9622247; doi:10.3201/eid2811.220029)
Supplement: Appendix — Additional information about invasive infections caused by Lancefield groups C/G and A Streptococcus, Western Australia, Australia, 2000–2018 [file 22-0029-Techapp-s1.pdf]

Article DOI: https://doi.org/10.3201/eid2811.220029

Invasive Infections Caused by Lancefield Groups C/G and A *Streptococcus*, Western Australia, Australia, 2000–2018

Appendix

Appendix Table 1. ICD-10-a.m. codes and PathWest panel codes for identification of invasive group C/G *Streptococcus* disease*

ICD-10-a.m. Principal Diagnosis Code†	Description
A40.8, with codes B95.41 or B95.42, without codes B95.0, B95.1 – B95.3, B95.48 – B95.8 or B96.1 – B96.8 as additional diagnoses	Other streptococcal sepsis
A48.3, with codes B95.41 or B95.42, without codes B95.0, B95.1 – B95.3, B95.48 – B95.8 or B96.1 – B96.8 as additional diagnoses	Toxic shock syndrome
G00.2, with codes B95.41 or B95.42, without codes B95.0, B95.1 – B95.3, B95.48 – B95.8 or B96.1 – B96.8 as additional diagnoses	Streptococcal meningitis
J15.4, with codes B95.41 or B95.42, without codes B95.0, B95.1 – B95.3, B95.48 – B95.8 or B96.1 – B96.8 as additional diagnoses	Streptococcal pneumonia‡
M00.2, with codes B95.41 or B95.42, without codes B95.0, B95.1 – B95.3, B95.48 – B95.8 or B96.1 – B96.8 as additional diagnoses	Other streptococcal arthritis and polyarthritis
M46.2, with codes B95.41 or B95.42, without codes B95.0, B95.1 – B95.3, B95.48 – B95.8 or B96.1 – B96.8 as additional diagnoses	Osteomyelitis of vertebra
M46.3, with codes B95.41 or B95.42, without codes B95.0, B95.1 – B95.3, B95.48 – B95.8 or B96.1 – B96.8 as additional diagnoses	Infection of intervertebral disc (pyogenic)
M46.5, with codes B95.41 or B95.42, without codes B95.0, B95.1 – B95.3, B95.48 – B95.8 or B96.1 – B96.8 as additional diagnoses	Other infective spondylopathies
M72.6, with codes B95.41 or B95.42, without codes B95.0, B95.1 – B95.3, B95.48 – B95.8 or B96.1 – B96.8 as additional diagnoses	Necrotising fasciitis
M86, with codes B95.41 or B95.42, without codes B95.0, B95.1 – B95.3, B95.48 – B95.8 or B96.1 – B96.8 as additional diagnoses	Osteomyelitis
P36.1, with codes B95.41 or B95.42, without codes B95.0, B95.1 – B95.3, B95.48 – B95.8 or B96.1 – B96.8 as additional diagnoses	Sepsis of newborn due to other unspecified streptococci
O85, with codes B95.41 or B95.42, without codes B95.0, B95.1 – B95.3, B95.48 – B95.8 or B96.1 – B96.8 as additional diagnoses	Puerperal sepsis
PathWest sample group	PathWest Panel code(s)
Blood	BC1, BC2, BS1
Tissue	BIO, SWT, TSI
Cerebrospinal fluid	CF2, FSL
Unspecified fluid	FLU (knee/hip aspirates, pleural fluid)
Peritoneal fluid	FSA
Amniotic fluid	FSB
Peritoneal dialysis fluid	FSC, SWS
Synovial fluid	FSJ
Pleural fluid	FSP, RTP
Placenta	PLA
Intraocular fluid	SWI
Vitreous fluid	SWV
Tips and lines	TPI

*ICD-10-a.m., International Classification of Diseases, Tenth Revision, Australian Modification.
†Note addition of code B95.41 and B95.42 (this had to be the first additional diagnosis) indicates Group C or G Streptococcus. The other B95 and B96 ICD codes indicate other bacterial codes and records were not eligible if these codes were present as a subsequent additional diagnosis due to resulting ambiguity as to the bacterial cause of invasive disease.
‡Note ICD-10 code J15.4 does not include pneumonia caused by group *B Streptococcus* and *Streptococcus pneumoniae.*

Appendix Table 2. Number of deaths and all-cause 30-d mortality for invasive group A and C/G *Streptococcus* disease, by age group*

	Group A (*1*)		Group C/G	
Age group in years	No. deaths (total)	% of deaths	No. deaths (total)	% of deaths
All age groups	116 (of 2,237)	5%	85 (of 1,270)	7%
0–<1	<5 (of 60)	-	0 (of 5)	0%
≥1–4	0 (of 91)	0%	0 (of <5)	0%
5–14	<5 (of 105)	-	0 (of 6)	0%
15–24	<5 (of 183)	-	<5 (of 52)	-
25–34	6 (of 292)	2%	<5 (of 80)	-
35–44	7 (of 380)	2%	<5 (of 133)	-
45–54	15 (of 342)	4%	7 (of 181)	4%
55–64	11 (of 259)	4%	7 (of 222)	3%
65–74	24 (of 218)	11%	16 (of 244)	7%
75–84	27 (of 200)	14%	19 (of 215)	9%
≥85	22 (of 90)	24%	32 (of 126)	25%
Missing/unknown	0 (of 17)		0 (of <5)	

*Cases with <5 are presented without accompanying percentage values to comply with confidentiality data requirements.

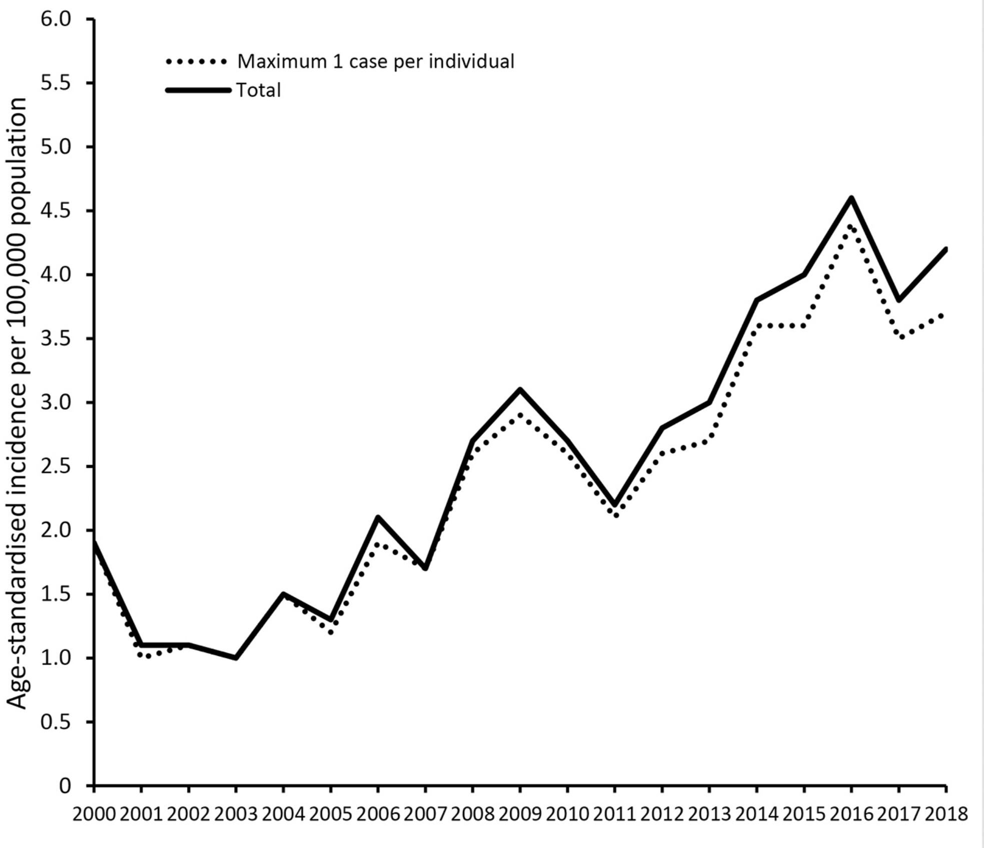

Appendix Figure 1. Age-standardized incidence of invasive group C/G *Streptococcus* disease, Western Australia, 2000–2018, with only 1 incident case allowed per person and allowing ≥1 case separated by at least 30 days.

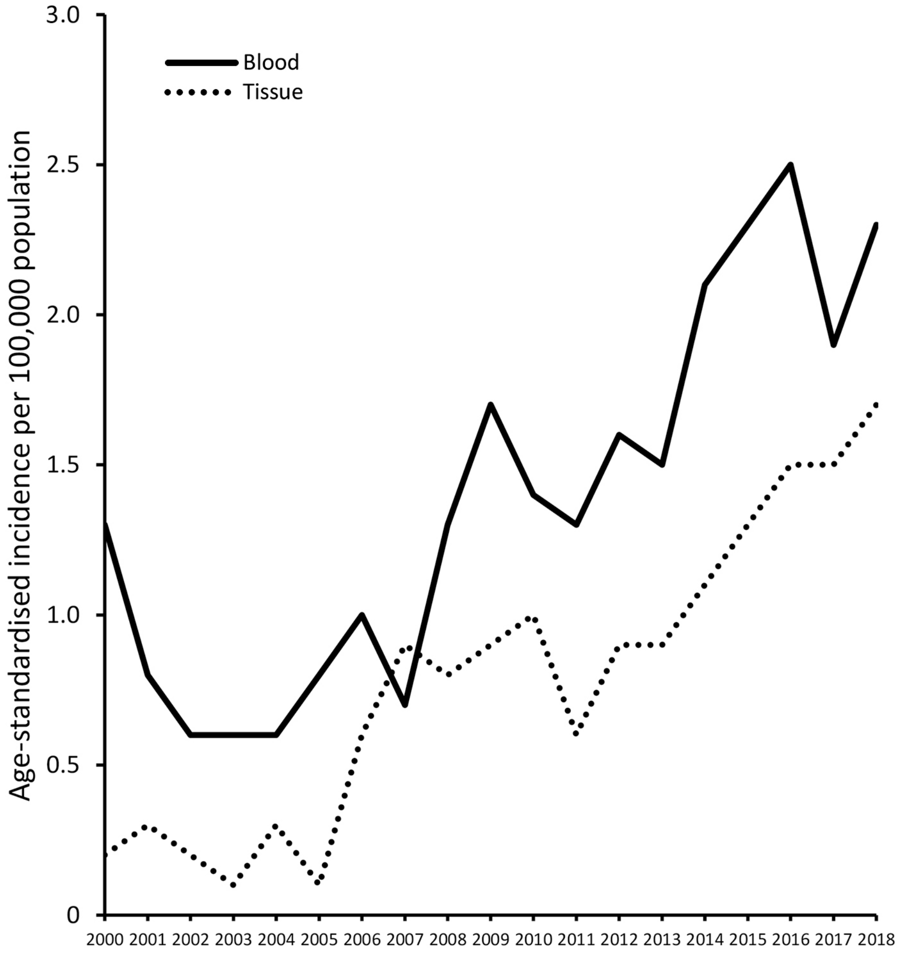

Appendix Figure 2. Age-standardized incidence of invasive group C/G *Streptococcus* disease with blood or tissue culture isolates, Western Australia, Australia, 2000–2018

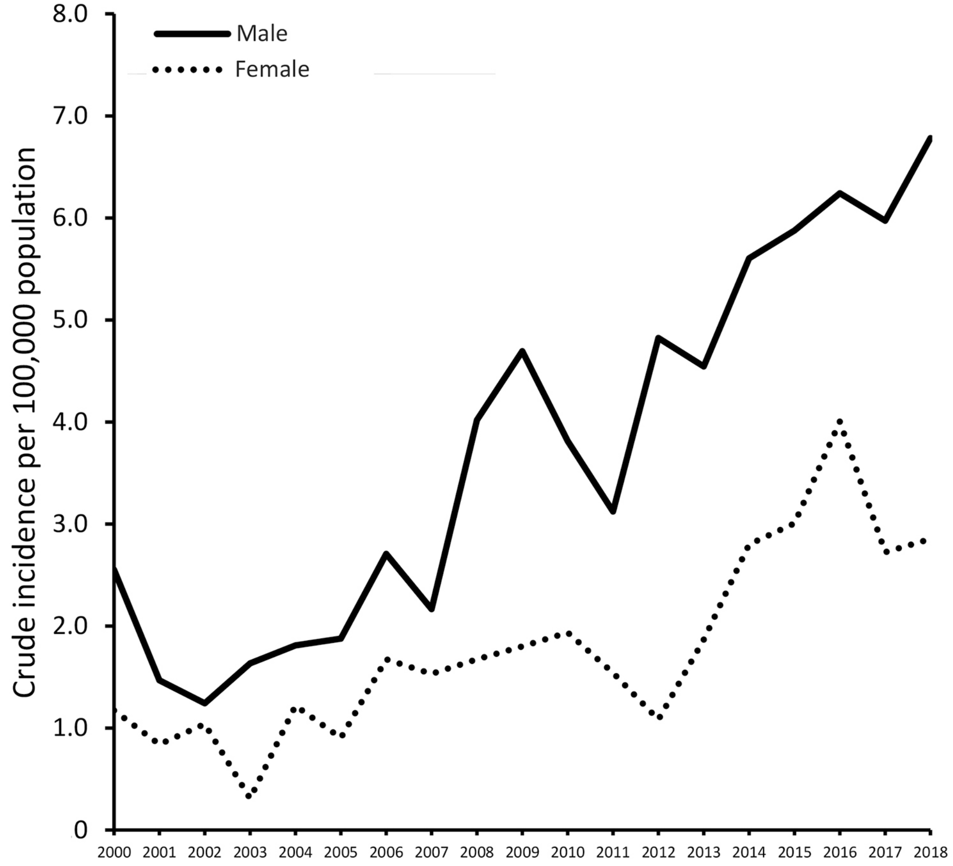

Appendix Figure 3. Crude incidence of invasive group C/G *Streptococcus* disease by sex, Western Australia, Australia, 2000–2018

References

1. Wright CM, Moorin R, Pearson G, Dyer JR, Carapetis JR, Manning L. Increasing incidence of invasive group A streptococcal disease in Western Australia, particularly among Indigenous people. Med J Aust. 2021;215:36–41. PubMed https://doi.org/10.5694/mja2.51117
